# Case Report: Case report: An unusual presentation of granulomatosis with polyangiitis

**DOI:** 10.12688/f1000research.133102.5

**Published:** 2023-10-23

**Authors:** Ichrak Bannour, Maroi Ben Brahim, Sondes Arfa, Soumaya ben Amor, Asma Ben Mabrouk, Olfa Berrich, Sonia Hammemi

**Affiliations:** 1 Laboratory of Molecular Immuno-Oncology, Faculty of Medicine, Universite de Monastir, Monastir, Monastir, 5000, Tunisia; 2 Immunology Laboratory, Fattouma Bourguiba Universitary Hospital, Universite de Monastir, Monastir, Monastir, 5000, Tunisia; 3 Biochemistry Laboratory, LR12ES05 LR-NAFS Nutrition-Functional Food and Vascular Health, Faculty of Medicine, Universite de Monastir, Monastir, Monastir, 5000, Tunisia; 4 Internal Medicine and Endocrinology Department, Tahar Sfar University Hospital, Universite de Monastir, Monastir, Monastir, 5111, Tunisia; 5 Department of Gastrology, Tahar Sfar University Hospital, Mahdia, Tunisia; 6 Internal Medicine and Endocrinology Department, Fattouma Bourguiba University Hospital, Universite de Monastir, Monastir, Monastir, 5000, Tunisia

**Keywords:** Granulomatosis with polyangiitis, Skin lesions, Necrotizing granulomatosis hepatitis, Case report.

## Abstract

**Aim:** We are reporting a case of an unusual presentation of granulomatosis with polyangiitis (GPA) with liver involvement.

**Case presentation:** A 45-year-old male patient presented with erythematous plaques on the face and bilateral nasal obstruction. On physical examination, the patient had a ring-shaped squamous plaque on the face. The laboratory findings revealed an accelerated erythrocyte sedimentation rate at 100 mm/h, an elevated C-reactive protein at 66 mg/L, hyper gamma globulinemia 16 g/L and an elevated alkaline phosphatase (twice the upper normal limit). The craniofacial and thoracoabdominal computed tomography (CT) -scans showed ethmoid and maxillary sinusitis, low facial bone density, multiple mediastinal and hilar lymphadenopathy, diffuse small pulmonary nodules, and hepatomegaly. A cutaneous lesion biopsy, the nasal mucosa, and the liver showed a chronic inflammatory granulomatosis process with necrosis. Serum anti-neutrophil cytoplasmic antibody (ANCA) against PR3 was positive. The clinical, biological, radiological, and histological findings substantiated the diagnosis of GPA. The patient received systemic steroids combined with cyclophosphamide pulses on days 1, 14 and 28 and then he was lost to follow-up. Two-years later, he presented with a cardiac failure and skin ulcer in the right lower limb. A nasal endoscopic exam showed nasal septum cartilage perforation with resorption of the middle and inferior nasal concha. Two weeks later, he developed a diffuse alveolar hemorrhage and was therefore transferred to the intensive care unit but died of respiratory failure 3 days later.

**Conclusion:** Clinicians should be aware of GPA atypical clinical manifestations.

## Introduction

Granulomatosis with polyangiitis (GPA) is an anti-neutrophilic cytoplasmic antibody (ANCA) associated systemic small vessel vasculitis with a necrotizing granulomatosis inflammation. It was described for the first time by Friedrich Wegener in 1936. ^1^ GPA has as a wide spectrum of clinical manifestations making it a challenging diagnostic dilemma for clinicians. The diagnosis is based on the association of clinical features, laboratory tests, and histological findings. The condition It is mainly characterized by necrotizing granulomatosis of the upper and lower respiratory tract and by glomerulonephritis. However, the granulomatosis inflammation or the vasculitis can affect any organ. Liver involvement in patients with GPA was rarely reported. We are reporting a case of an unusual presentation of GPA involving the upper airways, lungs, skin, and the liver with lethal cardiac manifestations.

## Patient and observation

### Patient

A 45-year-old Tunisian male patient working as a professor, who had no relevant family history presented with erythematous plaques on the face and bilateral nasal obstruction. He reported fatigue, loss of appetite, and unintentional weight loss with mouth and eye dryness for several months.

### Clinical findings

**Figure 1.**
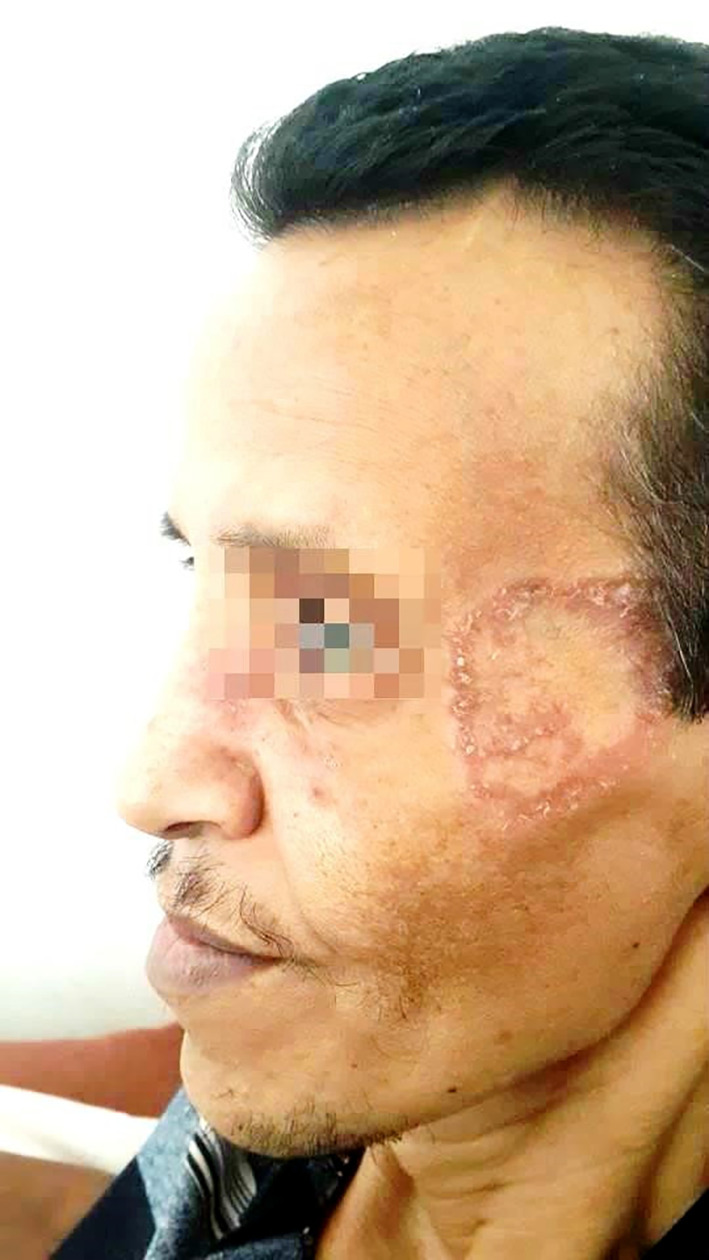
A ring-shaped squamous plaque on the face.

On physical examination, the patient had a ring-shaped squamous plaque on the face ( Figure 1) and unilateral submandibular lymphadenopathy. A nasal endoscopy found congested nasal mucosa with bloody crusts while laryngoscopy showed increased thickness of the right vocal cord and paralysis of the left vocal cord. The ophthalmological examination concluded a low tear break-up time. The pulmonary auscultation revealed bibasilar crepitations and abdominal palpation hepatomegaly. A biopsy was performed in the cutaneous lesion showing a granulomatosis inflammation. Nevertheless, the histopathological results of the minor salivary glands were normal.

### Diagnosis assessment

The laboratory findings showed an accelerated erythrocyte sedimentation rate (100 mm/h), an elevated C-reactive protein (66 mg/L), hyper gamma globulinemia (16 g/L), and an elevated alkaline phosphatase (twice the upper normal limit). The rest of the lab tests including cell blood count, creatinine, electrolytes, angiotensin-converting enzyme, aspartate aminotransferase, alanine aminotransferase and gamma-glutamyltransferase, GGT were normal. Urinalysis was negative for glucosuria, proteinuria, and blood cells. Serological tests for bacterial and viral infections including hepatitis B, hepatitis C were also negative. The PCR for M Tuberculosis and the QuantiFERON-TB Gold In-Tube (QFT-G) test for the diagnosis of tuberculosis disease were negative suggesting that there is not TB infection.

A nasal mucosa biopsy revealed a granulomatosis inflammation with necrosis.

**Figure 2.**
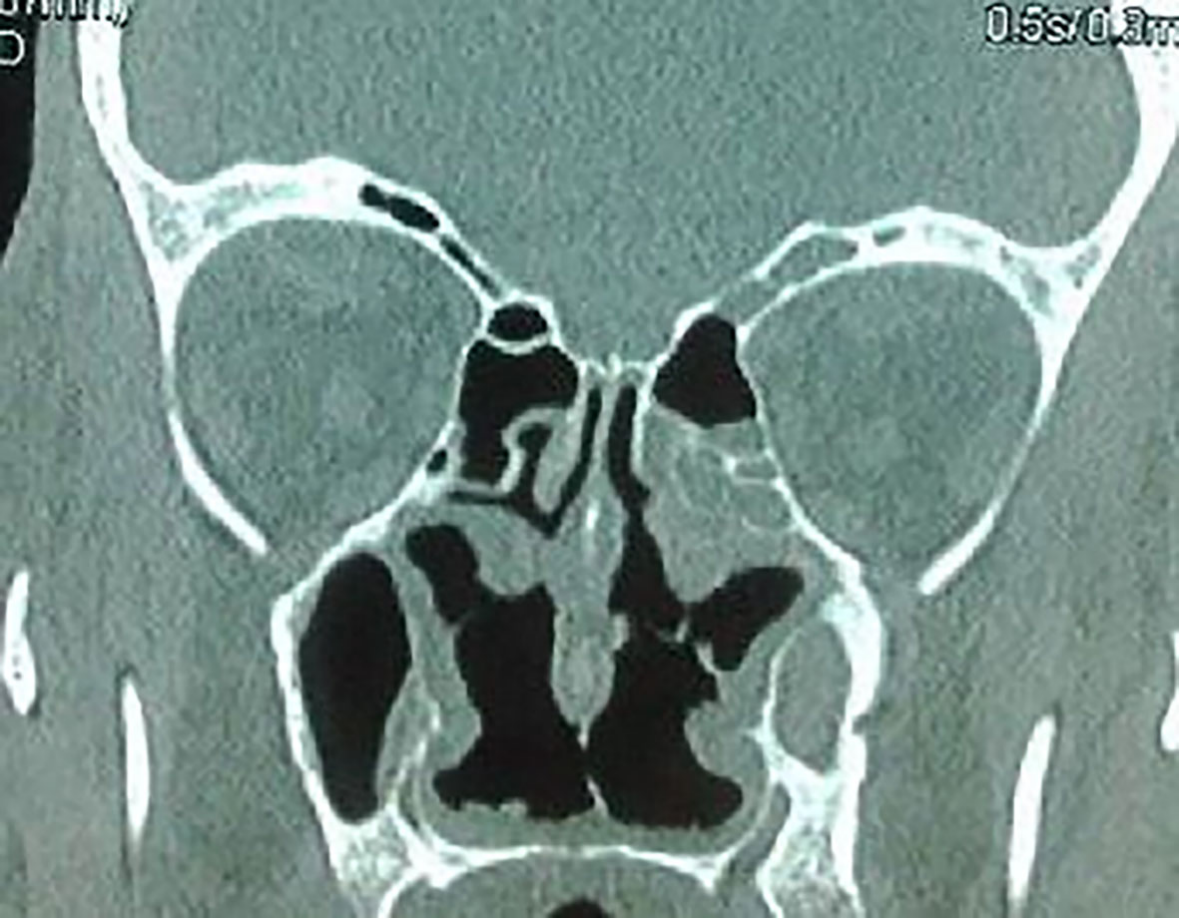
Ethmoid and maxillary sinusitis and low facial bone density.

The craniofacial and the thoracoabdominal computed tomography (CT) scans showed ethmoid and maxillary sinusitis, low facial bone density ( Figure 2), multiple mediastinal and hilar lymphadenopathy, diffuse small pulmonary nodules, peripheral, linear reticulations with subpleural cystic lesions, compatible with fibrosis, and hepatomegaly. Plethysmography showed a restrictive pattern with normal forced expiratory volume in 1 second/forced vital capacity (FEV1/FVC) ratio and severely diminished Total Lung Capacity (TLC of 64 per cent of the predicted level) and Forced Vital Capacity (FVC of 60 per cent of the predicted level). Autoimmune serology, including assays for auto-antibodies against nuclear antigen, SS-A/SS-B, anti-cyclic citrullinated peptide (anti-CCP), anti-ds-DNA was negative. Our patient had c-ANCA positivity with a titer of 1:40 (< 1:20). P-ANCA was negative. Proteinase 3 antibody (anti-PR3) was positive by an immunodot technique (Euroimmun, Germany). The serology was negative for autoimmune hepatitis and primary biliary cholangitis: effectively, Non-organ specific autoantibodies (NOSAs, tested by indirect immunofluorescence for antinuclear antibody (ANA), AMA (Antimitochondrial antibodies), SMA (Smouth Muscle Antibodies) and by immunoblotting for AMA type M2, liver kidney/microsome type 1 [LKM-1], LC1 (Liver Cytosol1 antibody), soluble liver antigen/liver pancreas [SLA/LP]) were negative. Gamma-globulin was at 0.81 (0.7-1.5) g/l; immunoglobulin-M, at 3.0 (0.46-3.04) g/l; and immunoglobulin-G, at 11.9 (7.51-15.6) g/l.

The clinical, biological, radiological, and histological findings substantiated the diagnosis of GPA.

Since liver involvement was suspected, a liver biopsy was also performed and was positive on H&E staining for chronic inflammatory granulomatosis process without necrosis.

### Therapeutic intervention

The patient received systemic steroids initiated by a 3-day regimen of methylprednisolone at a dose of 1 g a day, then oral prednisone at a dose of 1 mg/kg/day combined with cyclophosphamide pulses at a dose of 0.6 mg/m ^2^ on days 1, 14 and 28. Then he was lost to follow-up.

### Follow-up and outcome

**Figure 3.**
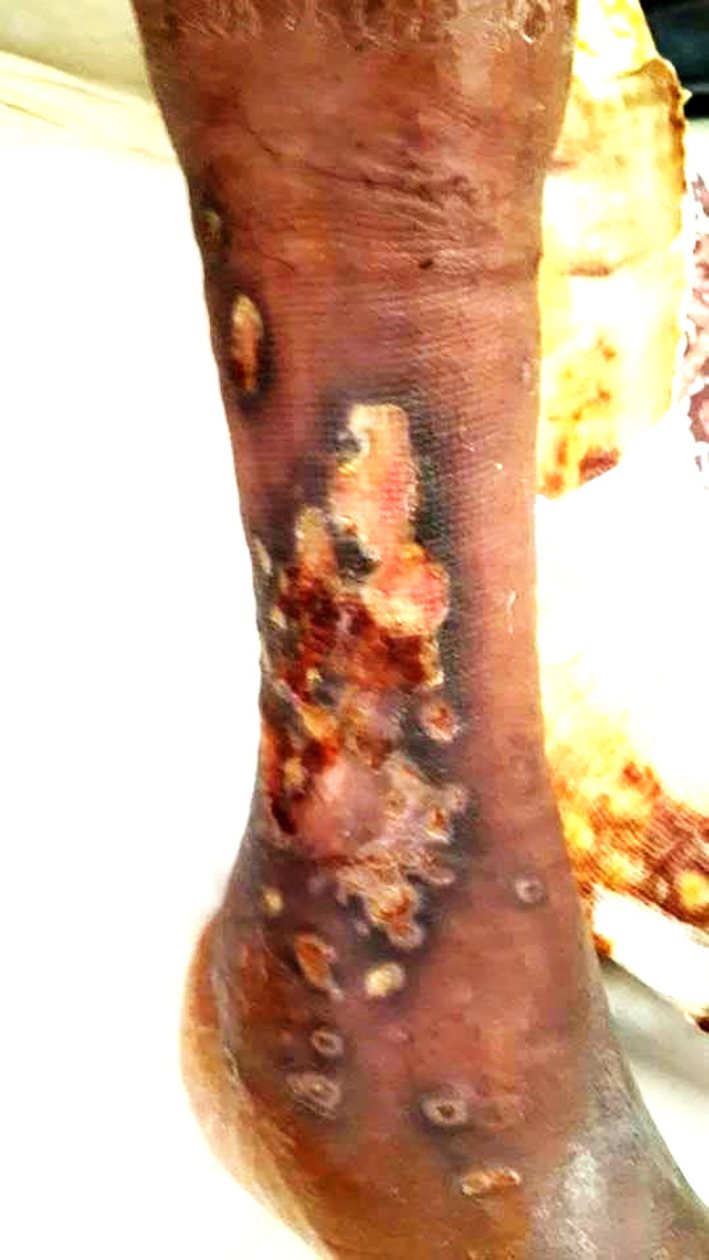
A large ulcer with necrosis and pus in the right leg.

Two years later, he was readmitted for dyspnea on exertion, oedema of the lower limbs, and a painful ulcer of his leg that had appeared 3 months prior to admission. On examination, he had tachypnea with a respiratory rate of 34 breaths/min, a large ulcer with necrosis and pus in the right leg ( Figure 3), and severe peripheral pitting oedema. The nasal endoscopic exam showed nasal septum cartilage perforation with resorption of the middle and inferior nasal concha. The lab tests revealed a biological inflammatory syndrome and elevated alkaline phosphatases. Pus culture was positive for *Pseudomonas aeruginosa.* A skin biopsy showed leukocytoclastic vasculitis. An electrocardiogram revealed a bifascicular block and a transthoracic echocardiography showed a dilated right ventricle, a pericardial effusion, a tricuspid valve regurgitation, and a pulmonary artery pressure of 80 mmHg. The patient received antibiotics to treat his skin infection and intravenous diuretics and oxygen *via* nasal cannula to manage his heart congestive failure. He was also started on three methylprednisolone pulses relayed by oral corticosteroids and cyclophosphamide. Two weeks later, he developed a diffuse alveolar hemorrhage. He was transferred to the intensive care unit, but died of respiratory failure 3 days later.

## Discussion

GPA is a severe life-threatening multi-systemic vasculitis usually involving the upper and lower respiratory tract and the kidneys. ^1^ However, it may involve other organs with variable clinical presentations.

Our patient initially presented with upper airways signs, pulmonary symptoms, skin lesions and liver involvement. The diagnosis of GPA was based on the combination of clinical manifestations with serological and histological evidence. ^1^

The liver involvement was confirmed by cholestasis and a chronic inflammatory granulomatosis process in the biopsy, which made this case worth reporting. In fact, although granuloma and/or the vasculitis may be found in any organ, the liver does not belong to the major target organs compared to the upper and lower airways. In fact, nasal and sinus involvement is the most common GPA manifestation. ^1^ Up to 85% of patients present with necrotizing granulomatosis lesions in the nasal cavity and sinuses. ^2^ The most frequent clinical features are nose crusting, blood stained rhinorrhea and nasal obstruction. ^3^ Pulmonary involvement is slightly less common and occurs in about 80% of patients with a wide spectrum of lesions. ^4^ Nodules are the most common radiological features. ^4^ Cavitation is a hallmark of the disease but it is only found in 22% of patients. ^4^ Liver involvement in patients with GPA is not quite clear. ^5^ It has been demonstrated that the liver is frequently affected in patients with active GPA when the affection is mirrored by liver test abnormalities. ^4^ The biochemical picture during active disease revealed both a cholestatic pattern ( *i.e.* increased g-GT and ALP) as well as a hepatocellular pattern (with increased ALT and AST) and was found in 2% to 25% patients with GPA at the time of diagnosis and was associated with a severe disease course and a poor prognosis. ^6^ However, necrotizing granulomatosis hepatitis in liver biopsy was rarely reported. ^4^ To the best of our knowledge, this might be the fifth case of hepatic involvement granulomatosis hepatitis reported in the literature. ^7^ All that cases were revealed by a persistent elevation of liver biological parameters and confirmed by liver biopsies after excluding adverse drug affects, viral hepatitis, alcoholic or ischaemic hepatitis. The serology was also negative for autoimmune hepatitis and primary biliary cholangitis, and the liver biopsies did not show evidence for these diseases. All patients in the literature unlike the patient in this case, responded to aggressive immunosuppressive drugs. 

Furthermore, it would be interesting to note that the patient presented with a squamous erythematosus plaque in the face as the initial presentation of the disease, which is particularly unusual since typical skin lesions in GPA consist of palpable purpura, papules, subcutaneous nodules and more rarely pyoderma-gangrenosum such as ulcers, digital ulcerations and gangrenes. ^1^^,^^4^ In fact, the cutaneous involvement in GPA is common and can be the revealing presentation in more than 30% of cases. ^8^ The spectrum of skin lesions is very large. ^1^ They can be classified as specific or nonspecific depending on the presence of histopathological evidence of vasculitis with or without granuloma. ^4^ Despite the unusual clinical presentation, the histological findings consisted of specific skin lesions in an ANCA associated vasculitis.

Mortality rate associated with GPA is as high as 80% in untreated patients. The conventional treatment for severe GPA consists of combination of high doses of systemic glucocorticoid and immunosuppressant agents which have contributed to the improvement of the prognosis of patients with GPA. However, these patients are still at risk of various complications. Several clinical factors were identified to predict higher mortality rates. In fact, liver involvement was proven to be related to the disease activity and to predict poor clinical outcomes. ^9^

In this case, the patient had pulmonary fibrosis PF complicated with pulmonary hypertension. PF is an uncommon manifestation observed in patients with ANCA vasculitis. ^10^ It is associated with worse prognosis.

In the present case, the patient did not comply with the treatment. He presented after two years with acute heart failure with a rapidly progressive fatal outcome. He had an acute congestive heart with concomitant pericarditis, dilated cardiomyopathy, valvular dysfunction and conduction defect which is extremely exceptional in this pathology. ^9^ Although cardiac involvement is considered to be uncommon in patients with GPA, the condition could be life threatening. ^6^^,^^8^ This manifestation is probably underestimated since it lacks specificity. ^11^ In fact, depending on the series and the methods applied such as electrocardiogram, echocardiography or cardiac magnetic resonance imaging (MRI), the prevalence of cardiac manifestations ranged between 40% and 60% of the patients. ^12^ Pericarditis is the most common cardiac manifestation and was reported in up to 35% of patients, followed by cardiomyopathy [30%] and coronary artery disease (12% of the patients). ^13^ Valve disease and conduction disorder including intraventricular conduction defects and atrioventricular blockage are less common. ^14^ As a result of aggressive treatment, cardiac involvement in patients with GPA was not associated with poorer outcome. ^14^

In this case the patient died from a respiratory failure due to diffuse alveolar hemorrhage which can be, either another rare and mortal complication of GPA occurring in only 5% to 10% of the patients, or a complication of the acute heart failure. ^15^ In another hand, necrotizing granulomatosis hepatitis could increase the risk of heart failure. The pathogenesis of death in this case might be multifactorial. GPA is associated with significant morbidity and mortality either due to irreversible organ dysfunction or due to the consequences of intensive/prolonged use of glucocorticoids or immunosuppressive agents.

Besides, the average life expectancy for a patient with GPA without any treatment is 5 months, with a 1-year survival rate of less than 30%. However, in recent times with the introduction of immunosuppressive agents and biologics, patients with GPA have a higher long-term survival rate and have been able to lead a relatively normal life. Patients with severe renal or cardiac involvement still have a guarded prognosis and a higher rate of mortality. ^16^

## Conclusion

The goal of presentation of this case was to present some rare and unusual manifestations of granulomatosis with polyangiitis presenting with hepatic granuloma and pulmonary fibrosis. Clinicians should be aware of liver involvement because it can be life-threatening. Systemic corticosteroids and immunosuppressant drugs remain the mainstay in the treatment of this potentially fatal disease. However, the frequencies of these atypical manifestations are to be studied in patients with GPA.

## Declarations

### Ethics approval

Not applicable.

### Consent for publication

Written informed consent was obtained from the patient’s next of kin for publication of this case report and any accompanying images.

## Authors’ contributions

Ichrak Bannour, Marwa Ben Brahim, OlfaBerriche, Sondes Arfa, Asma Ben Mabrouk and Sonia Hammemi all contributed equally to the conception, design, drafting and revising of the manuscript. All authors read and approved the final manuscript.

## Data Availability

All data underlying the results are available as part of the article and no additional source data are required.
