# Supplementary material for: Case Report: Case report: An unusual presentation of granulomatosis with polyangiitis
Source: F1000Res. 2023 Oct 23;12:430. Originally published 2023 Apr 21. [Version 5] doi: 10.12688/f1000research.133102.5 (PMC10600508; doi:10.12688/f1000research.133102.5)
Supplement: Peer review history

# Reviewer report: Reviewer response for version 5

**DOI:** 10.5256/f1000research.157826.r217613

**Published:** 2023-10-25

**Authors:** Meriem Kallel

**Affiliations:** 1 Immuno-Rheumatology Research Laboratory, Rheumatology Department, La Rabta Hospital, University of Tunis-El Manar, Tunis, Tunisia; 2 Immunology, La Rabta Hospital, Tunis, Tunisia

The authors reponded to all comments.

Are enough details provided of any physical examination and diagnostic tests, treatment given and outcomes?

Partly

Is the case presented with sufficient detail to be useful for other practitioners?

Yes

Is sufficient discussion included of the importance of the findings and their relevance to future understanding of disease processes, diagnosis or treatment?

Yes

Is the background of the case’s history and progression described in sufficient detail?

Yes

Reviewer Expertise:

Autoimmune diseases

I confirm that I have read this submission and believe that I have an appropriate level of expertise to confirm that it is of an acceptable scientific standard.

# Reviewer report: Reviewer response for version 4

**DOI:** 10.5256/f1000research.153391.r206976

**Published:** 2023-10-17

**Authors:** Meriem Kallel

**Affiliations:** 1 Immuno-Rheumatology Research Laboratory, Rheumatology Department, La Rabta Hospital, University of Tunis-El Manar, Tunis, Tunisia; 2 Immunology, La Rabta Hospital, Tunis, Tunisia

- The patient had mouth and eye dryness for several months: How did authors ruled out Sjogren’s syndrome? Pulmonary fibrosis may be related to this disease.
- Clarify the meaning of “The serology was negative for autoimmune hepatitis and primary biliary cholangitis”. Which autoantibodies were tested?
- The patient had elevated PAL and normal GGT: Could this be related to a bone and not hepatic origin?
- To rule out tuberculosis, authors mentioned only Quantiferon test which is not enough to confirm that the patient had not tuberculosis. Which  other tests were carried out mainly on biopsies (PCR?).  
- The patient had  a worse Prognosis: more reported data  about prognosis factor of granulomatosis with polyangiitis have to be mentioned in the discussion.

Are enough details provided of any physical examination and diagnostic tests, treatment given and outcomes?

Partly

Is the case presented with sufficient detail to be useful for other practitioners?

Yes

Is sufficient discussion included of the importance of the findings and their relevance to future understanding of disease processes, diagnosis or treatment?

Yes

Is the background of the case’s history and progression described in sufficient detail?

Yes

Reviewer Expertise:

Autoimmune diseases

I confirm that I have read this submission and believe that I have an appropriate level of expertise to confirm that it is of an acceptable scientific standard, however I have significant reservations, as outlined above.

# Reviewer report: Reviewer response for version 3

**DOI:** 10.5256/f1000research.152226.r188590

**Published:** 2023-07-20

**Authors:** Gloria Vasquez

**Affiliations:** 1 Rheumatology Section, Facultad de Medicina, Universidad de Antioquia, Medellín, Antioquia, Colombia

I approved the new version.

 Only have one suggestion, please edit this typewriting mistake:

 "The goal of presentation of this case was to present some rare and unusual manifestations of granulomatosis with polyangiitis presenting with hepatic granuloma and pulmonary fibrosis pulmonary fibrosis".

Are enough details provided of any physical examination and diagnostic tests, treatment given and outcomes?

Partly

Is the case presented with sufficient detail to be useful for other practitioners?

Partly

Is sufficient discussion included of the importance of the findings and their relevance to future understanding of disease processes, diagnosis or treatment?

Partly

Is the background of the case’s history and progression described in sufficient detail?

Partly

Reviewer Expertise:

Rheumatologist and immunology

I confirm that I have read this submission and believe that I have an appropriate level of expertise to confirm that it is of an acceptable scientific standard.

# Response

**Published:** 2023-07-20

**Authors:** Ichrak Bannour

**Affiliations:** Faculty of Medicine of Monastir, Tunisia

I do want to thank the reviewer Professor Gloria Vasquez for her corrections and suggestions of improvement. I corrected the typewriting mistake and omitted the expression "pulmonary fibrosis"

# Reviewer report: Reviewer response for version 2

**DOI:** 10.5256/f1000research.148656.r175860

**Published:** 2023-06-12

**Authors:** Gloria Vasquez

**Affiliations:** 1 Rheumatology Section, Facultad de Medicina, Universidad de Antioquia, Medellín, Antioquia, Colombia

I have reviewed the corrected version of the article, I realize that most of the suggestions were followed, but I still have doubts about the case and comments.

1. The cause of pulmonary hypertension described as “Showed a dilated right ventricle, a pericardial effusion, a tricuspid valve regurgitation, and a pulmonary artery pressure of 80mmHg” is not clear.
2. They describe an Immunodot technique to measure antiproteinase 3 levels, could you give the name of the brand?
3. In the discussion it is added that the heart failure of the patient is due to compromise by the disease, but the heart failure is right according to the echocardiography and associated with pulmonary hypertension, which is very rare.
4. Figure 3 is titled as a granulomatous lesion, but the granulomas are not shown or seen.
5. This sentence in the final conclusion is contradictory and I think that statement cannot be made "This case demonstrates that atypical clinical manifestations of GPA are very frequent".
6. I think it should be made clear that quantiferon, although it is positive in the presence of active tuberculosis, can also be positive in latent tuberculosis and is not a good diagnostic tool for active tuberculosis.

For all these reasons I still do not think the case is ready to be indexed.

Are enough details provided of any physical examination and diagnostic tests, treatment given and outcomes?

Partly

Is the case presented with sufficient detail to be useful for other practitioners?

Partly

Is sufficient discussion included of the importance of the findings and their relevance to future understanding of disease processes, diagnosis or treatment?

Partly

Is the background of the case’s history and progression described in sufficient detail?

Partly

Reviewer Expertise:

Rheumatologist and immunology

I confirm that I have read this submission and believe that I have an appropriate level of expertise to confirm that it is of an acceptable scientific standard, however I have significant reservations, as outlined above.

# Response

**Published:** 2023-06-27

**Authors:** Ichrak Bannour

**Affiliations:** Faculty of Medicine of Monastir, Tunisia

**Reviewer comments**

1. The cause of pulmonary hypertension described as “Showed a dilated right ventricle, a pericardial effusion, a tricuspid valve regurgitation, and a pulmonary artery pressure of 80mmHg” is not clear.

We thank the reviewer for underscoring this point which allows us to add that the patient had pulmonary fibrosis which could explain the pulmonary hypertension.   

 In fact, we added in line 41-47: that CT scan showed “peripheral, linear reticulations with subpleural cystic lesions, compatible with fibrosis. Plethysmography showed a restrictive pattern with normal forced expiratory volume in 1 second/forced vital capacity (FEV1/FVC) ratio and severely diminished Total Lung Capacity (TLC of  64 per cent of the predicted level) and Forced Vital Capacity (FVC of 60 per cent of the predicted level).” The causes of the pulmonary fibroses were screened “Autoimmune serology, including assays for auto-antibodies against nuclear antigen, SS-A/SS-B, anti-cyclic citrullinated peptide (anti-CCP), anti-ds-DNA, Rnase protection (RNP), sphingomyelin (SM), anti-Saccharomyces cerevisiae antibodies (ASCA IgA and ASCA IgG), was negative. “. And our patient had “c-ANCA positivity with a titer of 1:40 (< 1:20). P-ANCA was negative. Proteinase 3 antibody (anti-PR3) was positive by an immunodot technique.  The serology was negative for autoimmune hepatitis and primary biliary cholangitis.”

1. They describe an Immunodot technique to measure antiproteinase 3 levels, could you give the name of the brand?
  We are grateful to the reviewer for this comment. And we added in line 49 that the Immunodot technique to measure antiproteinase 3 levels was from (Euroimmun, Germany). 
2. In the discussion it is added that the heart failure of the patient is due to compromise by the disease, but the heart failure is right according to the echocardiography and associated with pulmonary hypertension, which is very rare.

We are grateful to the reviewer for this comment. The patient had lung fibrosis (probably due to his vasculitis) leading to a restrictive lung syndrome with pulmonary hypertension and right ventricular dysfunction. And he had also pericarditis which is the most common cardiac manifestation and was reported in up to 35% of patients.

3. Figure 3 is titled as a granulomatous lesion, but the granulomas are not shown or seen. We thank the reviewer for this comment. Unfortunately, we had not found a clear figure illustrating granuloma in the hepatic lesions. And as the case is very old I haven’t found the slides for proofreading. So I deleted the figure.

4.This sentence in the final conclusion is contradictory and I think that statement cannot be made "This case demonstrates that atypical clinical manifestations of GPA are very frequent".

 We thank the reviewer for underscoring this point which allows us to add that “The goal of presentation of this case was to present some rare and unusual manifestations of granulomatosis with polyangiitis presenting with hepatic granuloma and pulmonary fibrosis”.

5.I think it should be made clear that quantiferon, although it is positive in the presence of active tuberculosis, can also be positive in latent tuberculosis and is not a good diagnostic tool for active tuberculosis. We thank the reviewer for underscoring this point. The QuantiFERON(R) TB Gold (in Tube) assay is intended for use as an aid in diagnosis of TB infection. Negative results suggest that there is not TB infection. A positive test indicates that a person has been infected with TB bacteria. It does not tell whether the person has latent TB infection or has progressed to TB disease.

# Response

**Published:** 2023-06-30

**Authors:** Ichrak Bannour

**Affiliations:** Faculty of Medicine of Monastir, Tunisia

In this version we precised that pulmonary fibrosis is the cause of the pulmonary hypertension and the heart failure "a dilated right ventricle, a pericardial effusion, a tricuspid valve regurgitation, and a pulmonary artery pressure of 80mmHg". Second, we described the Immunodot technique used to measure antiproteinase 3 levels. Third, we deleted the figure 3 because we had not found a clear figure illustrating granuloma in the hepatic lesions. Than, we changed the conclusion and cited the aims of this case. Finally, we cited that Quantiferon can also be positive in latent tuberculosis and is not a good diagnostic tool for active tuberculosis.

# Reviewer report: Reviewer response for version 1

**DOI:** 10.5256/f1000research.146074.r170566

**Published:** 2023-05-12

**Authors:** Gloria Vasquez

**Affiliations:** 1 Rheumatology Section, Facultad de Medicina, Universidad de Antioquia, Medellín, Antioquia, Colombia

I consider that the case is very interesting, but there is data in the clinical history that are missing. In the initial evaluation of liver function, neither the levels of g-GT nor LA appear, in the presence of a granulomatous infiltration of the liver, these data are critical.

 In the image of the liver biopsy, it would be worth pointing out the findings and specifying the site of the taking, the staining and the resolution of the image in the figure caption.

 In the second part of the story, the patient arrives with right heart failure and pulmonary hypertension, its origin is not clear.

 The discussion refers to seven cases of liver involvement in the literature but does not describe whether the findings were similar to the case described or different.

 It is reported that liver involvement can be a cause of mortality but here the patient died of alveolar hemorrhage, so this aspect is not clear.

 It would be useful to describe the title of the anti PR3.

 The authors describe that tuberculosis was ruled out serologically, what type of test was used.

 I think that all these details should be clarified, the discussion should be improved, in order to take advantage of a case that is very interesting.

Are enough details provided of any physical examination and diagnostic tests, treatment given and outcomes?

Partly

Is the case presented with sufficient detail to be useful for other practitioners?

Partly

Is sufficient discussion included of the importance of the findings and their relevance to future understanding of disease processes, diagnosis or treatment?

Partly

Is the background of the case’s history and progression described in sufficient detail?

Partly

Reviewer Expertise:

Rheumatologist and immunology

I confirm that I have read this submission and believe that I have an appropriate level of expertise to confirm that it is of an acceptable scientific standard, however I have significant reservations, as outlined above.

# Response

**Published:** 2023-05-23

**Authors:** Ichrak Bannour

**Affiliations:** Faculty of Medicine of Monastir, Tunisia

**Reviewer comments**

In the initial evaluation of liver function, neither the levels of g-GT nor LA appear, in the presence of a granulomatous infiltration of the liver, these data are critical.

 We thank the reviewer for underscoring this point which allows us to add the initial evaluation of liver function in lines 31 and 33 “and an elevated alkaline phosphatase (twice the upper normal limit).” and “gamma-glutamyl transferase, GGT were normal”.

In the image of the liver biopsy, it would be worth pointing out the findings and specifying the site of the taking, the staining and the resolution of the image in the figure caption.

 We are grateful to the reviewer for this comment. We pointed out in the liver biopsy Liver biopsy hepatic sinusoids with neutrophilic infiltration and microabscess formation. And we added in line 48, 49 and 50 “Liver biopsy showing hepatic sinusoids with neutrophilic infiltration and microabscess formation (arrows), H&E staining, 200× magnification.”

**Figure 3. Chronic inflammatory granulomatosis process on biopsy: Liver biopsy showing hepatic sinusoids with neutrophilic infiltration and microabscess formation (arrows), H&E staining, 200× magnification.**

In the second part of the story, the patient arrives with right heart failure and pulmonary hypertension, its origin is not clear.

 We thank the reviewer for this remark. In this case the patient did not adhere to the immunosuppressive treatment and presented after two years with a rapidly lethal right heart failure. What made our patient different was the fact that he presented with an acute congestive heart with concomitant pericarditis, dilated cardiomyopathy, valvar dysfunction and conduction defect which is extremely exceptional. We precise in line 133-136 that “. He presented after two years with acute heart failure with a rapidly progressive fatal outcome. He had an acute congestive heart with concomitant pericarditis, dilated cardiomyopathy, valvular dysfunction and conduction defect which is extremely exceptional in this pathology”.

The discussion refers to four cases of liver involvement in the literature but does not describe whether the findings were similar to the case described or different.

 As underlined by the reviewer, this was the fifth case of liver involvement described in the literature all revealed by a persistent elevation of liver biological parameters and confirmed by liver biopsies after excluding adverse drug affect, viral hepatitis, alcoholic or ischaemic hepatitis in all patients. The serology was also negative for autoimmune hepatitis and primary biliary cholangitis, and the liver biopsies did not show evidence for these diseases. All patients the literature unlike the patient in this case, responded to aggressive immunosuppressive drugs. This was added in line 109-114: “All that cases were revealed by a persistent elevation of liver biological parameters and confirmed by liver biopsies after excluding adverse drug affects, viral hepatitis, alcoholic or ischaemic hepatitis. The serology was also negative for autoimmune hepatitis and primary biliary cholangitis, and the liver biopsies did not show evidence for these diseases. All patients in the literature unlike the patient in this case, responded to aggressive immunosuppressive drugs.”

It is reported that liver involvement can be a cause of mortality but here the patient died of alveolar hemorrhage, so this aspect is not clear.

 We thank the reviewer for this remark which allowed us to clarify the cause of mortality of the patient in this case. “In fact, he died from a respiratory failure due to diffuse alveolar hemorrhage which can be, either another rare and mortal complication of GPA occurring in only 5% to 10% of the patients, or a complication of the acute heart failure. In another hand, Necrotizing granulomatosis hepatitis could increase the risk of heart failure”. The pathogenesis of death in this case might be multifactorial. We added this in the discussion line 147-151.

It would be useful to describe the title of the anti PR3.

 We thank the reviewer for this interesting remark. Indirect immunofluorescence (IIF) for ANCA detection was performed using a commercial kit (Euroimmun®). Our patient had c-ANCA positivity with a titer of 1:40 (< 1:20). P-ANCA was negative. Positive serums for c- or p-ANCA at IIF are subsequently screened for antigenic specificity (MPO or PR3). Proteinase 3 antibody (anti-PR3) was positive by an immunodot technique (immunodot, Euroimmun(®). Then we added in line 38, 39 “Our patient had c-ANCA positivity with a titer of 1:40 (< 1:20). P-ANCA was negative. Proteinase 3 antibody (anti-PR3) was positive by an immunodot technique”

The authors describe that tuberculosis was ruled out serologically, what type of test was used.

 We thank the reviewer for this interesting remark. The QuantiFERON-TB Gold In-Tube (QFT-G) test for the diagnosis of tuberculosis disease was negative. We added this in line 37.
